# Effects of hyaluronic acid combined with anti-inflammatory drugs compared with hyaluronic acid alone, in clinical trials and experiments in osteoarthritis: a systematic review and meta-analysis

**DOI:** 10.1186/s12891-017-1743-6

**Published:** 2017-09-06

**Authors:** Thippaporn Euppayo, Veerasak Punyapornwithaya, Siriwadee Chomdej, Siriwan Ongchai, Korakot Nganvongpanit

**Affiliations:** 1 Animal Bone and Joint Research Laboratory, Department of Veterinary Biosciences and Public Health, Faculty of Veterinary Medicine, Chiang Mai University, Chiang Mai, 50100 Thailand; 2 Department of Food Animal Clinic, Faculty of Veterinary Medicine, Chiang Mai University, Chiang Mai, 50200 Thailand; 3 Department of Biology, Faculty of Science, Chiang Mai University, Chiang Mai, 50200 Thailand; 4 Department of Biochemistry, Faculty of Medicine, Chiang Mai University, Chiang Mai, 50200 Thailand; 5 Excellence Center in Veterinary Bioscience, Chiang Mai University, Chiang Mai, 50200 Thailand

**Keywords:** Anti-inflammatory drugs, Cartilage, Hyaluronic acid, Osteoarthritis, Systematic review

## Abstract

**Background:**

The objectives are to compare the efficacy of intra-articular hyaluronic acid (IA-HA) alone and in combination with anti-inflammatory drugs (IA-HA + AI), corticosteroids (CS) or non-steroidal anti-inflammatory drugs (NSAIDs) in clinical trials and in vivo and in vitro studies of osteoarthritis (OA).

**Methods:**

Data in the BIOSIS, CINAHL, Cochrane Library, EMBASE and Medline databases were collected and analyzed. Random effects models were used to compute the effect size (ES) of the mean difference in pain reduction scores from baseline and the relative risk (RR) of adverse events. The ES of histological scores in vivo and cartilage metabolism in vitro were also calculated. We conducted sensitivity analysis of blinding and intention-to-treat (ITT), compared IA-HA combined with CS vs. IA-HA alone in trials, and compared the effects of HA + AI vs. AI alone in vitro, including anabolic and catabolic gene expression.

**Results:**

Thirteen out of 382 papers were included for data analysis. In clinical trials, the ES of pain reduction scores within the 1st month was −4.24 (−6.19, −2.29); 2nd–12th month, −1.39 (−1.95, −0.82); and within one year, −1.63 (−2.19, −1.08), favoring IA-HA + AI (*P* < 0.001). The ES of RR was 1.08 (0.59, 1.98), and histological scores was 1.38 (−0.55, 3.31). The ES of anabolic gene expression was 1.22 (0.18, 2.25), favoring HA alone (*P* < 0.05); catabolic gene expression was 0.74 (−0.44, 1.53), favoring HA alone; and glycosaminoglycans remaining was −2.45 (−5.94, 1.03).

**Conclusions:**

IA-HA + AI had greater efficacy for pain relief than IA-HA alone within a one-year period. However, HA + AI down-regulated the *ACAN* gene when compared with HA alone in vitro.

## Background

At present, researchers around the world have concluded that osteoarthritis (OA), or degenerative joint disease, a major common joint disease in humans and animals, cannot be cured [1–5]. Although the cartilage cannot return to normal, some medications, such as corticosteroids (CS) and non-steroidal anti-inflammatory drugs (NSAIDs), are useful for relief of pain and inflammation in affected joints due to their inhibition of inflammatory cytokines [6, 7]. Intra-articular (IA) injection of CS (IA-CS) is allowed by the U.S. Food and Drug Administration (FDA) to reduce synovitis and effusion in OA [8], but NSAIDs are prohibited. Corticosteroids have potent anti-inflammatory effects by inhibiting phospholipase A_2_, reducing pain and effusion to a comparatively greater extent than NSAIDs, but prolonged use of CS may result in negative effects and accelerate OA progression. In in vitro studies, triamcinolone acetonide (TA) reduced glycosaminoglycan (GAG) synthesis and increased GAG degradation [9], and dexamethasone (DEX) induced chondrocyte apoptosis through activation of caspases and suppression of the Akt-phosphatidylinositol 3′-kinase signaling pathway [10].

Hyaluronic acid (HA) is a symptomatic slow-acting drug for osteoarthritis (SYSADOA) which is modestly effective in the treatment of moderate knee OA pain [11]. Since 1997, intra-articular injection of HA (IA-HA) in various preparations has been approved by the FDA for knee OA [12]. Previous studies have conducted a meta-analysis of published reports on the use of IA-HA in OA. In nearly all of the papers, HA had a greater effect on pain relief than a placebo [13–20]. Many recent reports have investigated the synergistic effects, drug interactions and decreased cytotoxicity of HA when combined with other drugs, in order to develop more effective OA treatments [21–27].

Much research has been conducted on HA combined with anti-inflammatory drugs (IA-HA + AI), via clinical trials and in vivo and in vitro studies. Some experiments have focused on the synergistic or antagonistic effects of these drug combinations. However, there is a lack of strong evidence in the literature supporting the efficacy of IA-HA + AI. This has led to the objective of this study: to analyze the effects of IA-HA + AI in clinical trials and in vivo and in vitro studies, using a systematic review and meta-analysis, to gain new insights that may help clinicians treat OA with IA-HA + AI with more confidence.

## Methods

### Literature search

Literature in the BIOSIS, CINAHL, Cochrane Library, EMBASE and Medline databases was included in a computerized search. We selected studies published in English that were performed from 1980 to 2016, using the following keywords: articular cartilage, chondrotoxicity, corticosteroid, degradation, hyaluronic acid, and NSAIDs. Reviewers screened titles, evaluated the eligibility of studies, and contacted the primary authors of abstracts with incomplete data. The results were compared and discussed to resolve any disagreements among the five reviewers (E.T., P.V., C.S., O.S. and N.K.).

### Selection

This study was categorized into three different parts: clinical trials, and in vivo and in vitro studies. Clinical trials consisted of randomized controlled trials that compared IA-HA and IA-HA + AI (CS or NSAIDs). This included IA-HA + AI administered under different conditions: i) injected together; ii) pre-injection with HA; or iii) post-injection with HA. The therapeutic effects of the drugs on OA joints in humans were assessed. In vivo and in vitro studies were those involving: i) normal cartilage; ii) OA-induced models; and iii) spontaneous OA in animals, focusing on the effects of drugs. Chondrocytes and cartilage with or without chemical- or cytokine-induced pathology were included. The positive or negative results of drugs in clinical trials and research experiments were subjected to data analysis. Duplicate articles, studies on systemic effects of drugs, review articles and case reports were excluded. To reduce bias, articles supporting commercial products were also excluded.

### Outcome measures

The outcome measures were different, depending on study type. For clinical trials, the primary outcome was pain relief from drugs recommended for OA [28]. The secondary outcome was adverse events (AE) that can occur after IA injection of drugs for OA [29]. All definitions of AE specified by the authors – such as pain, swelling, redness, heat, or loss of joint function typically related to IA drugs – were measured. For in vivo studies, data on histological scores were compared and analyzed. For in vitro studies, anabolic gene expression, i.e. aggrecan (*ACAN*) and collagen type II alpha 1 (*COL2A1*), catabolic gene expression, i.e. ADAM metallopeptidase with thrombospondin type 1 motif 5 (*ADAMTS5*), cyclooxygenase-2 (*COX-2*), interleukin 1 beta (*IL-1β*), matrix metalloproteinase-2 (*MMP2*), matrix metalloproteinase-3 (*MMP3*), and matrix metalloproteinase-13 (*MMP13*), and glycosaminoglycans (GAG) in chondrocyte pellets or cartilage explants were measured. For in vivo and in vitro experiments using various doses of HA or anti-inflammatory drugs, the highest dose in each study was used to calculate the effect size in order to avoid experimental bias.

### Data extraction

For the three study categories – clinical trials, and in vivo and in vitro studies – data on authors, study duration, mean outcome value, year of publication, and sample size were collected and recorded. Mean and standard deviation (SD) of data in graphs was estimated using the WebPlotDigitizer program. For clinical trials, we calculated the pain reduction from baseline of pain-related outcomes at all time points of each trial. We also generated new SD from the data on mean pain reduction scores from baseline over a one-year period (for all time points within one year, minus baseline). Moreover, we analyzed the data on mean pain reduction from baseline by groups: within the 1st month (all time points within the 1st month, minus baseline); and from the 2nd month to the 12th month (all time points during 2nd–12th month, minus baseline). We also analyzed the relative risk (RR) of AE after using IA-HA + AI compared with IA-HA alone. The data for each part of the analysis were pooled using random effects models. The effect size (ES) was calculated at a *P* value of less than 0.05 and reported as a 95% confidence interval (95% CI). The data were displayed in forest plots to illustrate the values for each part. The heterogeneity of data was presented as a percentage, based on the *I* ^2^ statistic. In this study, R version 3.2.3 was used for analysis of all data.

### Sensitivity analysis

Sensitivity analysis was performed for clinical trials and in vitro studies, but in vivo studies could not be analyzed because of different outcome measurements. Clinical trials were subgrouped into trials that reported blinding (single-blinding and double-blinding) and intention-to-treat (ITT) analysis (explicitly or not explicitly reported). Meta-regression analysis was used to assess the blinding of randomization and ITT. We directly compared the effects of IA-HA + CS with IA-HA alone. For in vitro studies, we separately analyzed the expression of the anabolic genes *ACAN* and *COL2A1*. Moreover, we evaluated the effects of HA + AI compared with each anti-inflammatory drug alone on anabolic gene expression, catabolic gene expression, and GAG remaining in chondrocyte pellets or cartilage explants.

## Results

### Trials and studies

**Fig. 1:**
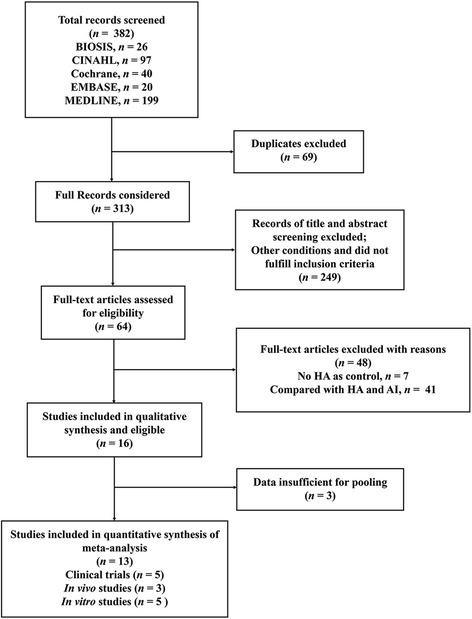
Flow diagram of articles to evaluate the systematic review and meta-analysis. AI, anti-inflammatory drugs; HA, hyaluronic acid; *n*, number of extracted papers

The research that was related to our scope included 382 papers. Duplicate papers (both duplicated in various databases or with the same or similar contents) were excluded (*n* = 69). Papers that did not fit the inclusion criteria (*n* = 249) were also excluded. Finally, the following 51 papers were excluded for reasons of: (1) not using HA as a control (7 papers); (2) studies of HA compared with anti-inflammatory drugs (41 papers); and (3) insufficient data for pooling (3 papers). Ultimately, 13 papers were found to be eligible and corresponded to inclusion criteria (Fig. 1). Twelve studies were published as original articles and one was published as a preliminary study.

Part I, clinical trials — Five trials [30–34] related to knee OA were included, with duration ranging from one week to one year. The average age range of patients was 40–80 years.

Part II, in vivo studies — Research related to the topic included three papers: in a rabbit OA model, studies on HA combined with liposomal celecoxib (CLX) [35], and HA combined with cortisone [36]; and in a rat OA model, cross-linked hyaluronic acid hydrogel (cHA gel) combined with DEX in surgery-induced OA [37].

Part III, in vitro studies — There were five studies [22, 23, 38–40], including anabolic gene (*ACAN* and *COL2A1*) expression, catabolic gene (*ADAMTS5*, *COX-2*, *IL-1β*, *MMP2*, *MMP3*, and *MMP13*) expression, and GAG remaining in chondrocyte pellets or cartilage explants.

The details of each study are shown in Table 1 for clinical trials, and in Tables 2 and 3 for in vivo and in vitro studies.

**Table 1:** Details of clinical trials in humans

Study	No. of patients in IA-HA	No. of patients in IA-HA + AI	Age (mean ± SD), years	Stage of OA^a^	Blinded experiment	Treatments	Outcome extracted	HA characteristics	Results
Grecomoro et al., 1992 [31]	20	20	42.3 ± 9.87	–	–	1. HA 20 mg, 5 weekly IA 2. DEX 0.4 mg at 1st IA followed by HA for 4 weekly IA	VAS	HA (MW 500–750 kDa)	• For IA-HA + DEX, pain intensity decreased more rapidly and had lower levels than IA-HA
Ozturk et al., 2006 [33]	24	16	HA, 58 ± 7.7 HA + TA, 58.06 ± 10.3	Grade 2–3 [65]	Single-blind	1. HA 30 mg, 3 weekly IA in 1st month and 3 IA in 6th month 2. TA 10 mg, prior to 1st and 4th IA of HA (at the same condition of HA injection above)	VAS, WOMAC pain	HA (MW 1000–2900 kDa)	• VAS and WOMAC pain scores were significantly reduced for IA-HA + TA • No significant differences in AE were reported between groups
Lee et al., 2011 [32]	22	21	HA, 69.1 ± 5.1 HA + KE, 61.1 ± 6.6	Grade 2–3 [65]	Single-blind	1. HA 25 mg, 5 weekly IA 2. KE 30 mg, 3 weekly IA, followed by HA, 2 weekly IA	VAS	HA (MW 940–1020 kDa)	• IA-HA + KE significantly improved pain compared with IA-HA alone • 5 of 21 patients had focal post-injection knee pain 8 h after IA
de Campos et al., 2013 [30]	52	52	HA, 61 ± 12 HA + TH, 65 ± 9	Grade 1–4 [66]	Double-blind	1. HA 48 mg, single IA 2. HA 48 mg and TH 20 mg, single IA	VAS, WOMAC pain	HA (MW 6000 kDa)	• At week 1, VAS and WOMAC pain scores were lower for IA-HA + TH when compared with IA-HA • There were no differences between groups at weeks 4, 12 and 24 • AE in each group were not different
Petrella et al., 2015 [34]	32	34	HA, 59 ± 12 HA + TA, 61 ± 11	Grade 2–3 [65]	Double-blind	1. HA (Hydros) 6 mL, single IA 2. TA 10 mg + HA (hydros) 6 mL, single IA	WOMAC pain	Hydros (modified HA polymer with a polyethylene glycol cross-linker which has the ability to entrap a low-dose corticosteroid)	• WOMAC pain scores were reduced in each group over 26 weeks • AE were similar in both treatment groups

^a^Classified by Kellgren & Lawrence grading system [65, 66]; −, no details

*AE* adverse events, *DEX* dexamethasone, *HA* hyaluronic acid, *IA* intra-articular injection, *KE* ketorolac, *kDa* kilodaltons, *MW* molecular weight, *OA* osteoarthritis, *TA* triamcinolone acetonide, *TH* triamcinolone hexacetonide, *VAS* visual analog scale, *WOMAC* Western Ontario and McMaster Universities Osteoarthritis Index

**Table 2:** Details of in vivo studies

Study	Species	No. of animals in IA-HA	No. of animals in IA-HA + AI	Treatments	Outcome extracted	HA characteristics	Results
Karakulum et al., 2003 [36]	Rabbit	7	7	1. HA 0.3 mL/day, IA at days 31, 38 and 45 2. MPA 15 mg/day IA at day 31 and HA 0.3 mL/day IA at days 38 and 45	Histological score; degeneration in cartilage zone [67]	HA (MW 1000–2900 kDa)	• Degeneration of cartilage decreased 72% for IA-HA and 88% for IA-HA + MPA at day 52
Dong et al., 2013 [35]	Rabbit	6	6	1. HA 0.3 mL (10 mg/mL), single IA 2. HA 10 mg/mL plus CLX 0.5 mg/mL for 0.3 mL, single IA	Global score; loss of cartilage layer, proteoglycan, and chondrocytes [68]	HA (MW 2.5 MDa)	• Single IA-CLX liposome or IA-HA alone did not significantly inhibit cartilage degeneration • IA-HA + CLX showed significant inhibition of cartilage degeneration
Zhang et al., 2016 [37]	Rat	10	10	1. IA-cHA gel, single IA 2. IA-cHA + Dex 0.2, 0.5 mg/mL gel, single IA	OARSI score; loss of proteoglycan [69]	Cross-linked HA hydrogel	• Quantification of histological findings: IA-cHA, 4.50 ± 0.87; IA-cHA + DEX, 3.00 ± 1.00

*CLX* celecoxib, *DEX* dexamethasone, *HA* hyaluronic acid, *IA* intra-articular injection, *cHA gel* cross-linked hyaluronic acid hydrogel, *kDa* kilodaltons, *MDa* megadaltons, *MW* molecular weight, *MPA* methylprednisolone, *OARSI* Osteoarthritis Research Society International

**Table 3:** Details of in vitro studies

Study	Species	No. of animals in HA	No. of animals in HA + AI	Treatments	Outcome extracted	HA characteristics	Results
Doyle et al., 2005 [38]	Horse	5	5	1. HA 0.1, 1, 1.5 mg/mL 2. MPA 0.05, 0.5, 5 mg/mL + HA 0.1, 1, 1.5 mg/mL	GAG in cartilage explants	HA (MW 500–730 kDa)	• Adding HA had little effect on MPA-induced cartilage matrix PG catabolism in articular cartilage explants
Yates et al., 2006 [40]	Horse	7	7	1. HA 0.2, 2 mg/mL 2. MPA 0.05, 0.5 mg/mL + HA 0.2, 2 mg/mL	*ACAN* expression GAG in chondrocyte pellets	HA (MW 500–730 kDa)	• *ACAN* expression was significantly reduced with MPA treatment • High concentration of MPA + HA increased GAG content in chondrocyte pellets
Schaefer et al., 2009 [39]	Horse	3	3	1. HA 0.5, 2 mg/mL 2. TA 0.06, 0.6 mg/mL + HA 0.5, 2 mg/mL	*ACAN*, *COL2A1* expression GAG in chondrocyte pellets	HA (MW 3,000,000 kDa)	• High concentration of HA and TA increased total GAG content • High concentration of HA or TA alone or in combination mitigated effects of IL-1 on GAG catabolism
Euppayo et al., 2015 [23]	Dog	3	3	1. HA 2.5 mg/mL 2. HA 2.5 mg/mL + CAR 6.2.5, 12.5, 25 mg/mL	*ACAN*, *COL2A1* expression	HA (MW 500–730 kDa)	• HA caused high expression levels of *COL2A1* and *ACAN* in OA cartilage • HA + CAR caused higher *COL2A1* and *ACAN* expression levels than CAR alone
Euppayo et al., 2016 [22]	Dog	2	2	1. HA 2.5 mg/mL 2. TA 0.09, 0.11 mg/mL + HA 2.5 mg/mL	*ACAN*, *COL2A1* expression	HA (MW 500–750 kDa)	• TA (0.11 mg/mL) up-regulated *ACAN* expression in canine OA chondrocytes • HA + TA did not show clearly beneficial effects

*ACAN* aggrecan gene, *CAR* carprofen, *COL2A1*, collagen type II alpha 1 gene, *GAG* glycosaminoglycans, *HA* hyaluronic acid, *kDa* kilodaltons, *MPA* methylprednisolone, *MW* molecular weight, *PG* proteoglycans, *TA* triamcinolone acetonide

### Clinical trials

#### Risk of bias in clinical trials

For clinical trials, we reviewed the risk of bias in five randomized trials. Two trials reported double-blinding [30, 34], two trials reported single-blinding [32, 33], and the other gave no details [31]. Three trials reported ITT analysis [30, 32, 34].

### Comparison between treatments

**Fig. 2:**
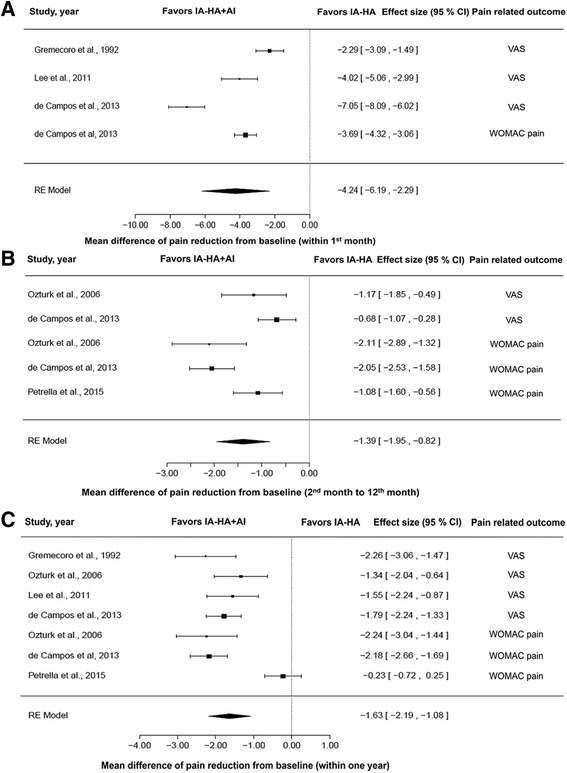
Forest plots of pain-related outcomes in clinical trials: mean difference in pain reduction from baseline within the 1st month (**a**), 2nd month to 12th month (**b**), and within one year (**c**). AI, anti-inflammatory drugs; CI, confidence interval; HA, hyaluronic acid; IA, intra-articular; RE, random effects model; VAS, visual analog scale; WOMAC, Western Ontario and McMaster Universities Osteoarthritis Index

For clinical trials, mean differences in pain reduction scores – based on the visual analog scale (VAS) and the Western Ontario and McMaster Universities Arthritis Index (WOMAC) pain score – within the 1st month, from the 2nd to 12th month, and within one year after injection were recorded in forest plots, as shown in Fig. 2a–c, respectively. In Fig. 2a, the effect size (ES) of the random effects model for the mean difference in pain reduction score within the 1st month was −4.24 (95% CI: −6.19 to −2.29, favoring IA-HA + AI; *P* < 0.0001). The *I* ^2^ value was 95.25%, indicating a substantial amount of heterogeneity. The ES of the random effects model for the mean difference in pain reduction score from the 2nd to 12th month was −1.39 (95% CI: −1.95 to −0.82; *P* < 0.0001). The *I* ^2^ value was 81.43% and the *P* value of heterogeneity was 0.0001, favoring IA-HA + AI (Fig. 2b). The ES of the random effects model for the mean difference in pain reduction score within one year was −1.63 (95% CI: −2.19 to −1.08, favoring IA-HA + AI; *P* < 0.0001). The *I* ^2^ value was 83.48%, with *P* value <0.0001 for the heterogeneity test (Fig. 2c). These results indicated that, in the first month after injection, using IA-HA + AI significantly reduced pain scores compared with IA-HA alone, by 4.24-fold (*P* < 0.0001). During the 2nd to 12th month, using IA-HA + AI significantly reduced pain scores compared with IA-HA alone, by 1.39-fold (*P* < 0.0001). Over a one-year period, using IA-HA + AI significantly reduced pain scores compared with IA-HA alone, by 1.63-fold (*P* < 0.0001).

### Safety of injection

**Fig. 3:**
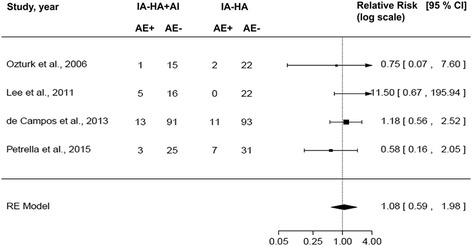
Forest plot of relative risk of AE after injection of HA or anti-inflammatory drugs. The size of boxes is proportional to the random effect weighted for the relative risk. AE, adverse events; AI, anti-inflammatory drugs; CI, confidence interval; HA, hyaluronic acid; IA, intra-articular; RE, random effects model

The RR of AE using IA-HA + AI compared with IA-HA alone in clinical trials is shown in Fig. 3. The ES of the random effects model in terms of RR was 1.08 (95% CI: 0.59 to 1.98; *P* = 0.80). The *I* ^2^ value was 0% and the *P* value of heterogeneity was 0.29. These results suggest that using IA-HA + AI was a related factor in RR, causing 8% more (1.08-fold) AE than IA-HA alone; however, this was not a significant difference.

### In vivo studies

**Fig. 4:**
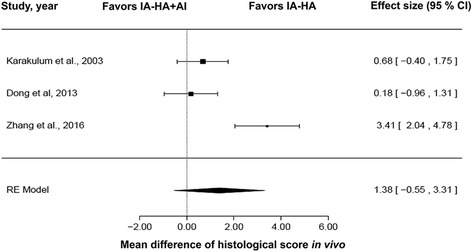
Forest plot of histological scores from in vivo studies. AI, anti-inflammatory drugs; CI, confidence interval; HA, hyaluronic acid; IA, intra-articular; RE, random effects model

Mean differences in histological scores are displayed in a forest plot in Fig. 4. The ES of the random effects model was 1.38 (95% CI: −0.55 to 3.31; *P* = 0.16) and the *I* ^2^ value was 87.35%, favoring HA. There was no significant difference between using IA-HA + AI and IA-HA alone when considering the severity of histological scores in OA animal models (*P* < 0.05).

### In vitro studies

**Fig. 5:**
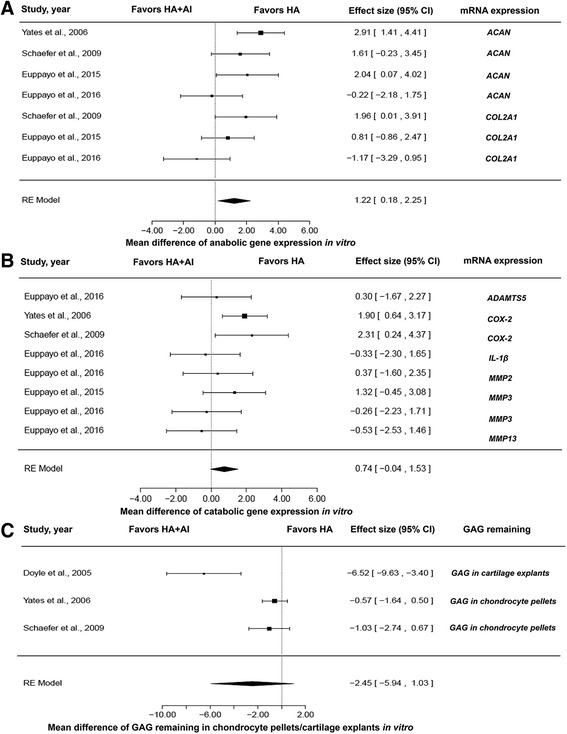
Forest plots of cartilage structure synthesis and degradation in vitro: (**a**) *ACAN* and *COL2A1* anabolic gene expression in chondrocytes or cartilage explants, comparing HA + AI and HA; (**b**) *ADAMTS5*, *COX-2*, *IL-1β*, *MMP2*, *MMP3*, and *MMP13* catabolic gene expression in chondrocytes or cartilage explants, comparing HA + AI and HA; (**c**) GAG remaining in chondrocyte pellets or cartilage explants, comparing HA + AI and HA. *ACAN*, aggrecan gene; AI, anti-inflammatory drugs; CI, confidence interval; *COL2A1*, collagen type II alpha 1 gene; GAG, glycosaminoglycans; HA, hyaluronic acid; RE, random effects model

Meta-analysis of cartilage structure synthesis and degradation is exhibited in Fig. 5. For anabolic gene expression (*ACAN* and *COL2A1*) (Fig. 5a), the ES of the random effects model was 1.22 (95% CI: 0.18 to 2.25, favoring HA alone; *P* = 0.0211). The *I* ^2^ value was 51.71% and the *P* value of heterogeneity was 0.038. The ES of catabolic gene expression (*ADAMTS5*, *COX-2*, *IL-1β*, *MMP2*, *MMP3*, and *MMP13*) was 0.74 (−0.04, 1.53), favoring HA alone (*P* = 0.0616) (Fig. 5b); *I* ^2^ value was 30.99% (*P* value of heterogeneity = 0.1992). For GAG in chondrocyte pellets or cartilage explants (Fig. 5c), the ES of the random effects model was −2.45 (95% CI: −5.94 to 1.03; *P* = 0.1678). The value of *I* ^2^ was 91.24% at *P* = 0.0019. These results suggested that HA + AI down-regulated anabolic gene expression when compared with HA alone (*P* < 0.05). HA alone induced catabolic gene expression more than HA + AI, but there was no significant difference. For GAG remaining in chondrocyte pellets or cartilage explants, there was no significant difference between HA + AI and HA alone.

### Sensitivity analysis

For clinical trials, we first confined pooled analysis to three trials that reported ITT analysis [30, 32, 34]. In this subset, the ES was −1.18 (95% CI: −2.15 to −0.22, favoring IA-HA + AI; *P* = 0.0165). The pooled analysis of trials that reported single-blind or double-blind methodology included four trials [30, 32–34]. The ES of this subset was −1.22 (95% CI: −1.93 to −0.51, favoring IA-HA + AI; *P* = 0.0008). Meta-regression analysis identified no significant difference between groups of blinding or ITT (*P* < 0.05). For the subgroup of IA-HA combined with CS vs. IA-HA alone, the ES was −1.38 (95% CI: −2.24 to −0.52, favoring IA-HA + CS; *P* = 0.0017). These results suggested that although we separately analyzed trials which reported only ITT or single/double-blinding, the results showed the same trends when interpreted for all five papers on clinical trials.

For in vitro studies, when comparing the effect of HA + AI vs. HA alone on *ACAN* expression, the ES was 1.67 (95% CI: 0.37 to 2.97, favoring HA alone; *P* = 0.0118); for *COL2A1* expression, the ES was 0.59 (95% CI: −1.09 to 2.27; *P* = 0.49). This meta-analysis showed that HA + AI had no clear effect on *COL2A1* expression when compared with HA alone. But when considering anabolic gene expression (including both *ACAN* and *COL2A1*) or in analyzing the subset of the *ACAN* gene alone, the results showed that HA + AI could reduce anabolic gene expression, especially the *ACAN* gene.

For the effect of HA + AI compared with AI alone on anabolic gene expression levels, the ES was −0.29 (95% CI: −1.04 to 0.45; *P* = 0.439). The ES of catabolic gene expression was 0.43 (95% CI: −0.21 to 1.07; *P* = 0.1897), while for GAG remaining in chondrocyte pellets or cartilage explants the ES was −2.6 (95% CI: −6.61 to 1.41; *P* = 0.2035). There was no significant difference between HA + AI and AI alone on anabolic or catabolic gene expression levels, or on GAG remaining in chondrocyte pellets or cartilage explants.

## Discussion

For clinical trials, the results of five papers [30–34] showed the same trend in pain relief. Intra-articular injection of NSAIDs (IA-NSAIDs) or IA-CS could act as rapid-onset pain relief – but not long (slow) acting as in the case of IA-HA – as observed from VAS or WOMAC pain scores within one year, especially at the earliest stage. High molecular weight HA (6000 kDa) was suggested for chronic knee OA pain because it relieved pain by week 12, while triamcinolone hexacetonide could relieve pain within 1 to 2 weeks [41]. Hence, using a combination of IA-HA + AI can be more effective for both short- and long-term pain reduction. This can be used either by pre-treating with anti-inflammatory drugs (IA-DEX or IA-TA) before a series of HA injections, or by administering in combination at the same time, e.g. IA-ketorolac (KE) combined with HA, or IA-TA combined with Hydros (modified HA polymer with a polyethylene glycol cross-linker), which can be entrapped by TA in its structure.

The reasons for collecting papers based on VAS and WOMAC pain scores, which were used as the main parameters for clinical trials, is because pain intensities were estimated by the patients themselves. The VAS, a unidimensional scale for measuring pain intensity, is the most frequently used pain rating scale [42] and is effective for determining average OA pain [43]; while the WOMAC pain subscale was included because it is widely used for measuring OA symptoms and physical disability status [44–47]. Other parameters, such as joint function, stiffness, and joint swelling, are also important in estimating clinical OA, but were not included in this study because of the limited amount of available data for pooling. Moreover, these parameters have no standard scoring system and are more subjective than the WOMAC pain score, so they should not be used for comparison between studies.

Although IA-AI and IA-HA have been widely used for treatment of OA joints, they could cause AE after injection. Overall RR results indicated that adding CS or NSAIDs in combination with IA-HA did not significantly increase AE compared with IA-HA alone. However, four of the studies included in this paper reported AE, such as severe pain and joint effusion [30, 32–34]; in these studies, the overall percentage of AE was 10.64% in the IA-HA group (20 of 188 patients) and 13.02% in the IA-HA + AI group (22 of 169 patients). It is possible that both IA-HA and IA-HA + AI can cause AE. However, adding AI in the same injection with HA or injecting the drugs before injection of HA did not increase AE in knee OA patients. Along with certifying the safety of IA-HA and IA-AI, the European Society for Clinical and Economic Aspects of Osteoporosis and Osteoarthritis (ESCEO) has recommended using IA-HA or IA-CS in symptomatic OA patients [48]. However, the Osteoarthritis Research Society International (OARSI) remains uncertain about recommending the use of IA-HA (except possibly for knee OA, as determined by physician/patient interaction), and has designated it as not appropriate for use in multiple-joint OA [49]. For administration of HA alone, for US-approved HA products for knee OA there was no significant difference in safety outcome and serious AE risk between IA-HA and a saline control [17]. However, AE of IA-HA included: mild transient local reaction; severe post-inflammation reactions which could occur due to administration of highly cross-linked high molecular weight HA [50]; immunogenic response [51]; or possibly a crystal-like response to large particles of HA [52]. For administration of IA-CS, infection, post-injection flare, crystal-induced synovitis, cutaneous atrophy and steroid arthropathy were noted as complications [52]. Moreover, steroid-induced (Charcot-like) arthropathy may occur after multiple injections [52].

In this study, we collected papers on HA combined with various types of AI that had different actions of anti-inflammation and analgesia. In step 2 of advanced pharmacological OA management, IA CS could be injected if a patient still had symptomatic OA [53]. A study comparing the effects of IA CS reported that TH was more effective than MPA on pain reduction at week 3, but that MPA resulted in a greater decrease in VAS and Lequesne index scores at week 8 of treatment [54]. TH was found to have a longer duration of action than TA for improvement of weight-bearing joints [55], while DEX produced analgesia similar to morphine in chronic arthritis [56]. Along with IA NSAIDs, there are a few of the previous study when compared with IA CS. KE is a classical NSAID that could be safely used for IA administration in post-operative pain relief [57, 58]. Some NSAIDs also had beneficial effects in in vivo and in vitro studies, e.g. IA CLX could suppress IL-1β, TNF, and MMP-3, and improved pathological changes of cartilage, similar to IA HA in a rabbit OA model [59]. Likewise, carprofen could decrease the severity of OA cartilage lesions in conjunction with decreasing the width of osteophytes in dog OA model [60]. Based on this study, IA NSAIDs may be used for clinical treatment of OA.

Degradation of aggrecan and collagen in cartilage structure is a manifestation of OA [53, 54]. When considering drug combinations in experiments, this research revealed that adding IA-CS or IA-NSAIDs in combination with HA may reduce the anabolic effect of HA on cartilage by down-regulating anabolic gene (*ACAN*) expression when compared with IA-HA alone. Hence, those using IA-HA + AI should be aware that AI may reduce anabolic gene expression levels. But these combinations had no clear effect on the level of catabolic gene expression and of GAG protein remaining in cartilage, or on histological changes [61, 62]. This may be due to the use of various histological grading criteria, different drug dosages and study durations, and a limited number of research reports. Further research should be undertaken to confirm these points.

In this study, we analyzed various MW of exogenous HA preparations, including low (MW 500–730 kDa), intermediate (MW 800–2000 kDa) and high MW (average: 6000 kDa) [63] and newly modified structures of HA to entrap other drugs. The effect of each MW of IA-HA + AI on OA joints should be collected, but there were a low number of papers for pooling data. Although we included combinations of HA with several different drugs, the results showed significantly reduced pain for the combinations compared with HA alone.

Limitations of this study are a low number of related research reports and small pooled sample size because this research was conducted based on a literature database search [64]. Moreover, in clinical trials of OA, the comparison of IA-AI and IA-HA + AI is interesting to include, but no conclusions can be drawn because of the low number of experiments using anti-inflammatory drugs alone to treat OA for an extended period. However, the strengths of this paper are revealing data on the use of HA plus AI in the field of clinical, in vivo and in vitro*.* To update the new knowledge of HA + AI based on increased population of humans and animals, using statistical analysis.

## Conclusions

Published data indicate with a good level of evidence that intra-articular injection of HA combined with anti-inflammatory drugs can potentially relieve pain in OA knee patients without increasing serious AE when combined with HA alone. However, in vitro studies indicate caution when using these in combination due to the potential reduction in expression levels of anabolic genes, especially *ACAN* expression which may encode aggrecan structure in cartilage.

## Abbreviations

*ACAN*: Aggrecan gene
AE: Adverse events
CAR: Carprofen
cHA gel: Cross-linked hyaluronic acid hydrogel
CI: Confident interval
CLX: Celecoxib
*COL2A1*: Collagen type II alpha 1 gene
CS: Corticosteroids
DEX: Dexamethasone
ESCEO: European Society for Clinical and Economic Aspects of Osteoporosis and Osteoarthritis
GAG: Glycosaminoglycan
HA: Hyaluronic acid
IA: Intra-articular injection
ITT: Intent-to-treat
kDa: Kilodaltons
KE: Ketorolac
MDa: Megadaltons
MPA: Methylprednisolone
MW: Molecular weight
NSAIDs: Non-steroidal anti-inflammatory drugs
OA: Osteoarthritis
OARSI: Osteoarthritis Research Society International
PG: Proteoglycans
RE: Random effect model
RR: Relative risk
SD: Standard deviation
TA: Triamcinolone acetonide
TH: Triamcinolone hexacetonide
VAS: Visual analog scale
WOMAC: Western Ontario and McMaster Universities Osteoarthritis Index
